# Comprehensive Open-Source Ecosystem for Raman and SERS Spectroscopy: Introducing SpectraGuru

**DOI:** 10.1021/acs.analchem.5c07799

**Published:** 2026-04-06

**Authors:** Fengbo Ma, Jiaheng Cui, Amit Kumar, Yanjun Yang, Xianyan Chen, Yiping Zhao

**Affiliations:** † School of Electrical and Computer Engineering, College of Engineering, 1355 The University of Georgia, Athens, Georgia 30602, United States; ‡ Department of Physics and Astronomy, The University of Georgia, Athens, Georgia 30602, United States; § Department of Epidemiology & Biostatistics, College of Public Health, The University of Georgia, Athens, Georgia 30602, United States

## Abstract

Raman and Surface-Enhanced Raman Scattering (SERS) spectroscopy are powerful techniques for molecular identification and characterization, yet widespread adoption is often limited by the lack of accessible, standardized tools for preprocessing, analysis, and data management. Here, we introduce SpectraGuru, an open-source web-based platform designed to provide a comprehensive ecosystem for Raman and SERS research. SpectraGuru integrates a modular workflow for data upload, interactive preprocessing, advanced statistical analysis, and visualization, all accessible through a user-friendly browser interface. The platform’s key contributions include a fully open-source, web-based system; comprehensive preprocessing and analysis tools covering tasks such as interpolation, despiking, baseline correction, normalization, peak identification, and multivariate methods like hierarchical clustering, Principal Component Analysis (PCA), and t-distributed stochastic neighbor embedding (t-SNE); and an integrated PostgreSQL database that supports FAIR (Findable, Accessible, Interoperable, Reusable) storage of spectra and metadata to enhance reproducibility and data sharing. Demonstrations with experimental data sets showcase SpectraGuru’s ability to transform raw spectra into clean, interpretable data and to reveal meaningful patterns across diverse analytes. By addressing challenges in preprocessing standardization, database integration, and analytical flexibility, SpectraGuru aims to accelerate spectral research and foster community-driven development in Raman and SERS spectroscopy.

## Graphical abstract

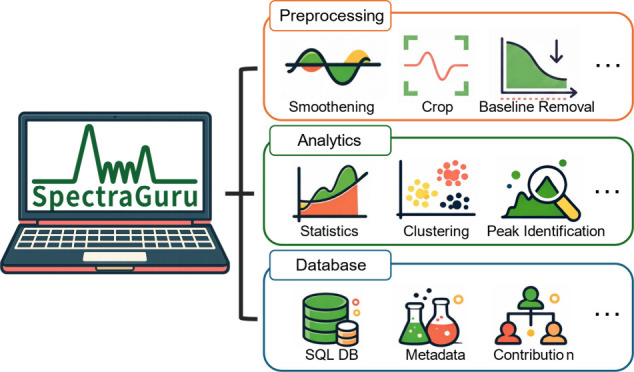

## Introduction

Raman and Surface-Enhanced Raman Scattering (SERS) spectroscopy are powerful analytical techniques with broad applications in molecular identification, characterization, and detection.^1^ However, extracting meaningful information from raw spectra remains a nontrivial task, as preprocessing steps such as baseline correction, noise filtering, normalization, and peak detection are critical for accurate interpretation and reproducibility.^2,3^ Despite these needs, the current landscape is fragmented, with diverse tools and a lack of open, publicly accessible databases, limiting the scalability and reproducibility of spectroscopic workflows.^4^

A first major challenge is the absence of standardized frameworks for preprocessing and spectral data management.^5^ Researchers often resort to manually scripting pipelines in Python or MATLAB, leading to a proliferation of fragmented, lab-specific codebases.^6^ Although these scripts typically implement common preprocessing and analytical algorithms, they often use inconsistent parameters and lack adequate documentation. Such variability undermines reproducibility and makes cross-study benchmarking extremely difficult.^7^

Second, while a wide range of spectroscopic software packages exists, most impose restrictions that prevent open and integrated workflows. Commercial suites such as Horiba LabSpec,^8^ Thermo Fisher’s OMNIC,^9^ Renishaw’s WiRE,^10^ and Tec5USA’s MultiSpec^11^ are distributed exclusively with vendor hardware and rely on native, undocumented file formats (e.g., .ngs, .spa/.spg, .wdf). Although allowing export to general file types, their core pipelines still privilege proprietary data structures, preventing seamless exchange with tools or community platforms. Stand-alone or vendor-agnostic applications offer partial flexibility but introduce new technical and licensing constraints. For example, StellarNet’s StellarPro^12^ is tied to proprietary hardware protocols; Spectragryph^13^ uses local SQLite libraries incompatible with community databases; Bio-Rad’s KnowItAll^14^ relies on a closed schema and licensing model that restrict transparent data exchange; and eigenvector Research’s PLS_Toolbox^15^ operates as a licensed add-on to another licensed computational environment. Across this fragmented landscape, no platform currently offers an open, community-maintained architecture that couples reproducible preprocessing with scalable machine learning pipelines. Recent open-source and academic tools, such as OpenSpecy^16^ and RamApp,^17^ have made important steps toward improving accessibility through lightweight, browser-based interfaces, but still lack comprehensive databases and standardized data management frameworks. RamanSPy^18^ is an open-source package but lacks a user interface, which creates a barrier for researchers who are not familiar with Python setup process. These platforms often lack advanced algorithms, centralized data storage, or robust analytic capabilities such as clustering, classification, and dimensionality reduction.^19^ As a result, there is no open-access system that integrates preprocessing, machine learning, and centralized data management in a reproducible, modular, and community-extensible manner.

Parallel efforts in Raman and SERS spectral data repositories remain equally fragmented. Commercial collections such as the S.T. Japan Spectra Database,^20^ Bio-Rad’s KnowItAll Raman Library,^21^ and Thermo Fisher’s Omnic Specta library^22^ provide large holdings but are costly, locked to proprietary formats, and offer limited user’s right for data mining or machine-learning applications.^19^ Despite their scale, these resources do not implement FAIR (Findable, Accessible, Interoperable, Reusable) principles, limiting their utility for modern algorithm-driven research. Open-access databases add breadth but remain narrowly scoped: RRUFF focuses on minerals;^23^ Raman Open Database^24^ and Romanian Database of Raman Spectroscopy^25^ likewise target crystallography and mineral species; Specarb^26^ curates carbohydrate spectra; and the Raman Spectroscopic Library concentrates on historical pigments.^27^ These initiatives often rely on static web interfaces without harmonized metadata schemas, which limits interoperability and large-scale analysis. Even community-led initiatives like Project Raman by Li et al.^28^ demonstrate the value of shared efforts but highlight the ad hoc nature of current practices, falling short of enabling interoperable, scalable infrastructures. Across both commercial and open repositories, the absence of FAIR-aligned design principles prevents the development of the interoperable, extensible, and scalable infrastructures needed to support the next generation of Raman and SERS data analytics.

To address these critical gaps, we present SpectraGuru, an open-source browser-based platform that establishes a standardized and reproducible workflow for Raman and SERS data analysis. Built on a web-based application framework, SpectraGuru requires no local installation and integrates preprocessing, machine learning, and centralized database management into a seamless interface. Users can upload spectra or query data directly from the integrated repository, apply preprocessing such as baseline correction, smoothing, and normalization, and visualize results in real time. Analytical units include statistics, clustering, classification, dimensionality reduction, and spectral decomposition, enabling complete end-to-end workflows within a unified environment. Beyond serving as a multifunctional analysis tool, SpectraGuru is designed as the foundation of a FAIR-aligned open-source ecosystem for the spectroscopy community. Its modular architecture supports transparent algorithm development, standardized metadata handling, and scalable database expansion. The platform promotes reproducibility, interoperability, and community participation by allowing researchers to share, annotate, and reuse data sets under consistent processing standards. Through its integration of data management, visualization, and analytical capabilities, SpectraGuru transforms isolated spectral analyses into a collaborative, data-driven environment, empowering users to accelerate discovery, validate results across laboratories, and contribute to the collective advancement of Raman and SERS spectroscopy.

## Architecture, Interface, and Data Management Framework

SpectraGuru introduces three principal contributions to the spectroscopy and machine learning communities shown in Figure 1: a spectral database, multiple spectral preprocessing functions, and basic spectral analytics tools. It offers the following advantages:

**Figure 1:**
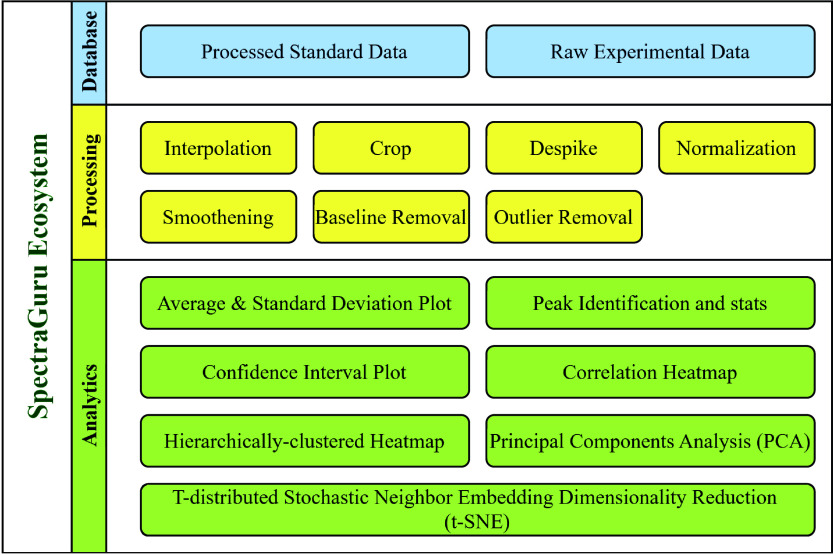
Overall pipeline for the SpectraGuru ecosystem.

### Standardization of Preprocessing Workflows

By consolidating common techniques within a modular, version-controlled platform, SpectraGuru promotes consistency, transparency, and reproducibility across projects.

### Unified End-to-End Capability

SpectraGuru integrates preprocessing, analytical computation, and database components into a unified browser-based architecture to enable continuous data flow across all stages of the spectroscopy workflow.

### Centralized Spectral Database

The relational database enforces rich metadata standards, supports community-driven data contributions, and enables the structured storage and retrieval of both raw and processed spectra. This resource facilitates reproducible research, machine learning model development, and collaborative innovation in spectroscopy.

The first stable version of SpectraGuru was publicly released at spectraguru.org on November 11, 2024, followed by an updated version on November 1, 2025. The open-source codebase is freely available on GitHub (https://github.com/FengboMa/SpectraGuru_beta). As of late 2025, the platform has been accessed by over 27,498 unique IP addresses, accounting for 537,532 visits from 85 countries and regions worldwide, reflecting its rapidly growing global reach and community engagement. In addition to the hosted web application, SpectraGuru can be deployed locally on institutional servers or personal computers using the publicly available source code. This deployment flexibility provides users with full control over computational resources, eliminates the reliance on external network connectivity, and enables secure offline operation when required.

In the following sections, we outline the design principles, system architecture, and functionalities of SpectraGuru. We demonstrate its application across representative case studies, compare its performance to existing tools, and discuss its potential as a sustainable resource for the Raman and SERS community.

## System Overview and User Interface

SpectraGuru is a modular platform developed in Python using the Streamlit framework^29^ to support interactive, browser-based applications. Its design emphasizes standardization through a unified DataFrame representation for all spectra, ensuring consistency across preprocessing, analysis, and visualization. The platform integrates with widely used scientific libraries, including NumPy,^30^ pandas,^31^ SciPy,^32^ scikit-learn,^33^ matplotlib^34^ and seaborn,^35^ and provides a foundation for reliable data manipulation, statistical analysis, and visualization. A detailed summary of the preprocessing and analysis features implemented in SpectraGuru, including their functionality and source of implementation, is provided in Table S1 in Supporting Information Section S1.

The user interface follows a consistent two-panel layout (Figure 2). The left sidebar provides structured navigation and parameter control. Figure 2a illustrates the main navigation menu, allowing users to switch seamlessly among application units such as Data Upload, Processing, and Analytics. Figure 2b shows the parameter adjustment section within the Processing unit, where users can select specific preprocessing steps including interpolation, smoothing, normalization, and outlier removal and then execute or reset these operations through clearly labeled action buttons. The main workspace enables interactive data visualization and control. Figure 2c displays the spectrum selection interface, where users can choose specific spectral data sets to visualize and activate fast-mode plotting when needed. Figure 2d presents the interactive spectral plot, which dynamically renders Raman spectra in real time and supports user interactions, such as mouse hover tooltips, zooming, and data inspection for individual samples. Data export and plot export functionalities are provided at the bottom of the page, allowing users to conveniently save both processed data and visual outputs for further analysis or publication.

**Figure 2:**
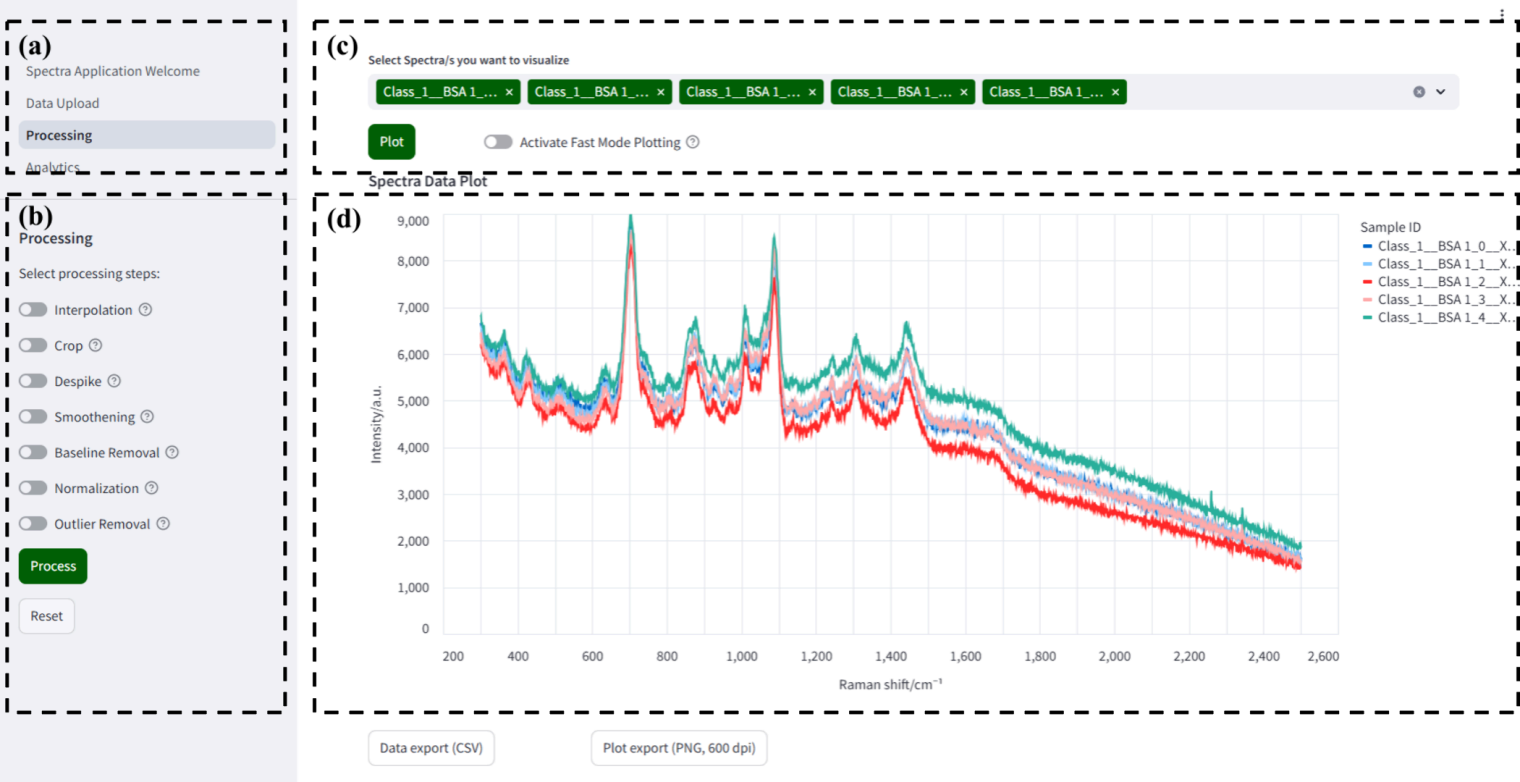
Graphical user interface of the processing unit in SpectraGuru. The sidebar panel is divided into two sections: (a) navigation controls for switching between modules and (b) parameter adjustment widgets, which adapt dynamically to the selected function (illustrated here with preprocessing tools). The right-hand side contains the main workspace, including (c) plot controls for spectrum selection and visualization triggers and (d) the interactive visualization panel, which supports real-time rendering, mouse tooltips, and direct interaction with spectral data.

This layout enables users to modify parameters in the sidebar while observing immediate changes in the main display, facilitating both exploratory analysis and systematic workflows. By combining modular design with interactive feedback, the interface balances clarity for new users with the flexibility needed for advanced research applications.

### Preprocessing

Preprocessing in SpectraGuru is implemented as a dedicated and modular workflow stage that provides a comprehensive set of tools for transforming raw spectra into consistent, analysis-ready data. The available operations include baseline correction, smoothing, despiking, normalization, cropping, interpolation, and outlier detection. Baseline correction methods include polynomial fitting,^36,37^ adaptive iteratively reweighted penalized least-squares (airPLS),^38,39^ and Gaussian–Lorentzian fitting.^40,41^ Noise reduction can be performed using Savitzky-Golay filtering^42^ or Fourier transform filtering.^43^ Normalization options such as area normalization, peak normalization, and min/max scaling standardize spectra for comparison across samples. Interpolation and cropping functions ensure that spectra can be aligned and restricted to the relevant wavenumber regions. Outlier detection identifies anomalous spectra by comparing each spectrum against correlation, standard deviation, and distance thresholds, which together help remove signals arising from the experimental artifacts.

Recent studies have explored parameter-free or data-driven baseline correction strategies based on deep learning architectures, including convolutional autoencoders^44^ and triangular deep convolutional networks,^45^ as well as adaptive optimization of classical penalized least-squares methods such as asPLS^46^ and OP-airPLS.^39^ While these adaptive methods reduce the need for manual parameter tuning, they still rely on algorithm-specific optimization procedures and careful validation to ensure reliable performance. In addition, deep learning approaches generally require large, well-curated training data sets, rigorous validation protocols, and careful cross-instrument generalization testing. Such requirements remain nontrivial for heterogeneous Raman and SERS data sets collected across laboratories with varying substrates, acquisition parameters, and signal-to-noise characteristics.

For these reasons, fully parameter-free automation has not yet been integrated into the current production version of SpectraGuru. Instead, the platform emphasizes transparent, user-guided preprocessing combined with explicit parameter tracking, which mitigates hidden algorithmic bias while preserving analytical flexibility. Default parameter values derived from commonly adopted practices in the literature are provided to assist users in establishing reasonable starting points. Importantly, all user-selected preprocessing steps and parameter values are automatically recorded and associated with the corresponding spectral data set, ensuring that workflows remain fully transparent, reproducible, and auditable.

All preprocessing functions are presented with interactive controls, and any adjustments are reflected immediately in the visualization panel. This design can reduce the iterative trial-and-error cycle common in offline analysis. Each operation can be executed independently, and identical preprocessing steps can be applied simultaneously to hundreds of spectra loaded into the application. Processed spectra and intermediate results can be exported in standard formats such as csv for downstream applications.

### Analytics

The analytics unit provides methods for statistical analysis, clustering, dimensionality reduction, and supervised classification. Available tools include group statistics (means, variances, and confidence intervals), correlation heatmaps, and peak identification functions.^32^ Dimensionality reduction methods such as principal component analysis (PCA)^47^ and t-distributed stochastic neighbor embedding (t-SNE)^48^ facilitate the exploration of high-dimensional spectral data sets. Clustering techniques, including hierarchical clustering^49^, support the grouping of similar spectra. Adjustable parameters within each method allow the analyses to be tailored to different experimental data sets.

### Database Design

A data management framework forms the foundation of the SpectraGuru ecosystem, ensuring that spectral data are handled in a standardized, reproducible, and FAIR^50^ compliant manner. The workflow begins with a dedicated Data Upload unit, which serves as the entry point for incorporating new experimental spectra or retrieving existing data sets from the integrated SpectraGuru repository. This design allows users to seamlessly combine novel data with standardized reference spectra while maintaining consistent metadata and processing standards. Supported data formats for manual data upload are summarized in Supporting Information Section S2, with illustrative examples of the two-column spectrum data structure provided in Figure S1. These guidelines define acceptable file types and data structure requirements. Spectral data uploaded for analysis remain under user control, and long-term storage in the shared database occurs only when explicitly selected by the user, allowing flexible data management consistent with institutional and research requirements. This user-controlled storage model ensures transparency in data retention while allowing contributors to decide whether their spectra remain private for individual analysis or are shared to support community-wide reuse.

The integrated database provides a robust and scalable infrastructure for storing spectral data and their associated metadata. Implemented in PostgreSQL for its reliability and extensibility, the architecture is organized around two principal tables: one for raw data records, which capture individual experimental spectra across replicates and conditions, and another for standard data, which store processed, averaged spectra representing canonical samples from experimental batches. This schema maintains a clear distinction between unprocessed and curated data sets, supporting data traceability and reproducibility.

Comprehensive metadata is a cornerstone of the database design. Each entry is annotated with key attributes describing analyte identity and condition, Raman/SERS experimental conditions, and spectrum acquisition parameters—elements essential for ensuring scientific reproducibility and enabling complex multiparameter queries. As illustrated in Figure 3, the workflow integrates experimental data ingestion, metadata annotation, and preprocessing steps that organize spectra into raw and standard data tables within the PostgreSQL framework. The current database includes 3082 spectra covering diverse analyte types, including chemicals,^51^ biomarkers,^52^ viruses,^40,53,54^ bacteria,^41,55^ dyes, proteins, DNA, RNA,^56^ and experimental controls. Details of database implementation are provided in Supporting Information Section S3. The structured metadata schema and field definitions are summarized in Table S2, and the database size and composition, including standardized and raw spectral data records, are summarized in Tables S3 and S4. Uploaded spectra in the database are assumed to have been precalibrated by the user when instrument response correction is required. Metadata fields are provided to document the calibration status, instrument configuration, and acquisition parameters to ensure transparency and traceability.

**Figure 3:**
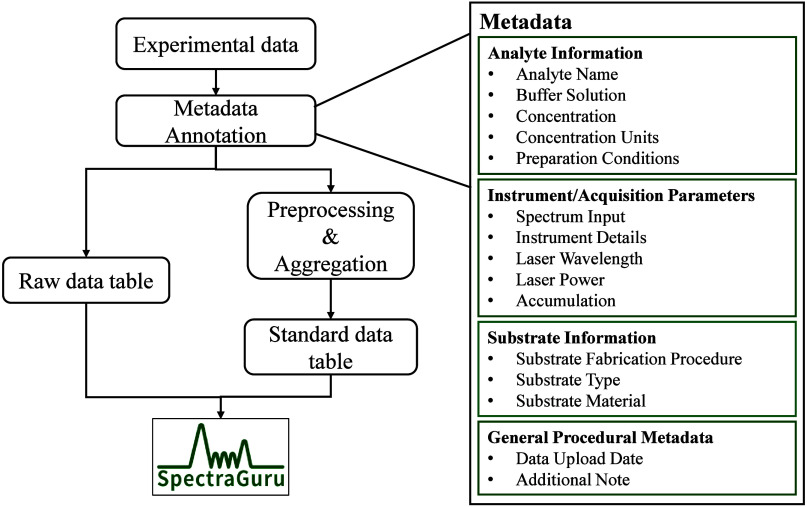
Schematic representation of the SpectraGuru database workflow.

## System Evaluation and Results

In this section, we demonstrate major features of SpectraGuru using real experimental data to evaluate the system’s performance across its core functionalities, including preprocessing steps and analytical tools.

### Preprocessing Steps

To demonstrate the preprocessing capabilities of SpectraGuru, we applied the full preprocessing workflow to five raw Bovine Serum Albumin (BSA) spectra (Figure 4a), which displayed prominent cosmic-ray spikes, unsmoothed profiles, and extended featureless regions. Within the current server configuration, SpectraGuru can preprocess hundreds of spectra in parallel.

**Figure 4:**
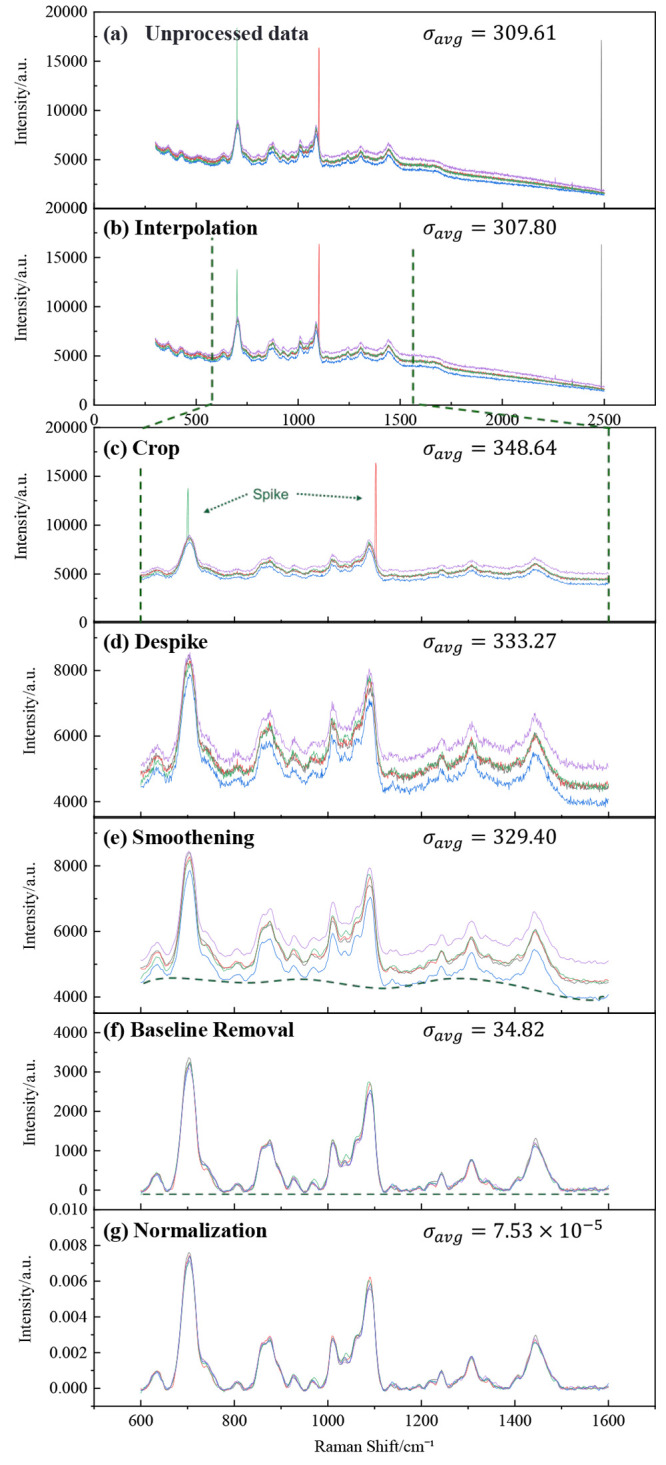
Sequential preprocessing steps applied to BSA spectral data: (a) raw BSA spectra with cosmic-ray spike; (b) interpolation applied and Raman shifts rounded to the nearest integer; (c) spectral range cropped to the feature-rich region (600 to 1600 cm^–1^); (d) manual identification and removal of spike regions at 700 cm^–1^ and 1100 cm^–1^; (e) Savitzky-Golay smoothing applied with window length = 15 and polynomial order = 2; (f) baseline corrected using the airPLS algorithm with λ = 100, polynomial order = 1, maximum number of iterations = 15, and convergence tolerance = 0.001; (g) area normalization applied to yield the final processed spectrum. Data were processed with SpectraGuru and then replotted using Origin Pro 2018b.

To quantitatively assess the quality of each preprocessing step, we defined an average spectral variation, denoted σ_*avg*_, which measures the overall variability. For a set of N spectra, let *k* index the wavenumber positions ranging from 1 to *M* across the spectral axis. The average spectrum at each wavenumber is given by *I̅*_*SERS*_(*Δv*_*k*_) (Definition provided by eq S1 in Supporting Information Section S4). Then the average spectral variation is defined as

$$\sigma_{avg}=\frac{1}{M}\overset{M}{\underset{k=1}{\sum }}\sqrt{\frac{1}{N-1}\overset{N}{\underset{i=1}{\sum }}{\left(I_{i}\left(\Delta v_{k}\right)-{\mathrm{I̅}}_{SERS}\left(\Delta v_{k}\right)\right)}^{2}},k=1,2,...,M,$$ 1

where *I*_*i*_(*Δv*_*k*_) is the intensity of the *i*-th spectrum at wavenumber *Δv*_*k*_. The initial σ_*avg*_ value for the five raw BSA spectra was 309.61.

The workflow began with interpolation (Figure 4b), rounding wavenumbers to the nearest integer to standardize spacing and create consistent data points across the spectra. Cropping (Figure 4c) was then manually applied to limit the spectral range to 600–1600 cm^–1^, removing regions without relevant features. Despike (Figure 4d) followed, using a manual despike function to target spikes identified within the 600–750 cm^–1^ and 1050–1150 cm^–1^ ranges, with a detection threshold of 300 and a window length of 11 to identify and correct these artifacts. Smoothing (Figure 4e) was performed by using a Savitzky-Golay filter with a window length of 15 and a polynomial order of 2, which reduced high-frequency noise while preserving important spectral features. Across these initial steps, σ_*avg*_ remained within 300–350, because interpolation, cropping, despiking, and smoothing modify either the spectral range or only local spectral intensities without affecting the overall global statistics.

A major change occurred after baseline removal (Figure 4f), implemented using the adaptive iteratively reweighted penalized least-squares (airPLS) algorithm with λ = 100, polynomial order = 1, a maximum of 15 iterations, and a convergence tolerance of 0.001. Baseline trends were effectively suppressed, causing the five spectra to collapse onto one another; and σ_*avg*_ dropped dramatically to 34.82, nearly an order of magnitude reduction. The corresponding relative variation $\frac{\sigma_{avg}}{\mathrm{integrated spectral intensity}}\approx 7.93\times 10^{-5}$ highlights the impact of baseline removal as a global preprocessing step that strongly influences spectral statistics. The dashed lines in both parts e and (f) illustrate the underlying baseline present prior to and after correction. Finally, area normalization (Figure 4g) was applied to scale each spectrum so that the integrated area equaled one, enabling direct spectral shape comparison across samples. After normalization, σ_*avg*_ further decreased to 7.53 × 10^–5^, and the relative variation also equaled 7.53 × 10^–5^, indicating that the spectra are tightly collapsed and highly consistent after all preprocessing steps.

Overall, this sequence of operations demonstrates SpectraGuru’s ability to transform raw Raman spectra into clean, standardized, and analysis-ready data sets suitable for quantitative or machine-learning-based downstream analysis.

### Analytics Steps

After completing the preprocessing steps to upload spectra, users can navigate to the Analytics unit to perform more analyses. Detailed implementation procedures and mathematical formulations for each analytical method are provided in the Supporting Information Section S4, eqs S1–S13. The following subsections introduce the analytics tools available in SpectraGuru, each presented under its respective paragraph title.

#### Peak Identification and Statistics

The SciPy peak-prominence method is well suited for Raman analysis because it measures how strongly a peak stands out from its surrounding valleys, making it insensitive to noise, sampling density, and baseline fluctuations. By evaluation of the vertical distance between each peak and its nearest trough, the algorithm can reliably distinguish true spectral features from minor variations or overlapping signals. This method was applied to the average BSA spectrum after baseline removal and area normalization. The algorithm automatically detected five prominent peaks at 702, 877, 1087, 1308, and 1443 cm^–1^ as marked in Figure 5a. For comparison, running the same procedure on the average unprocessed spectrum (Figure 5b) produced peaks at 704, 875, 1087, 1308, and 1441 cm^–1^. The slight wavenumber shifts for the processed and unprocessed spectra illustrate how preprocessing influences peak position identifications. Both panels also support interactive exploration, allowing users to hover over each peak for additional information.

**Figure 5:**
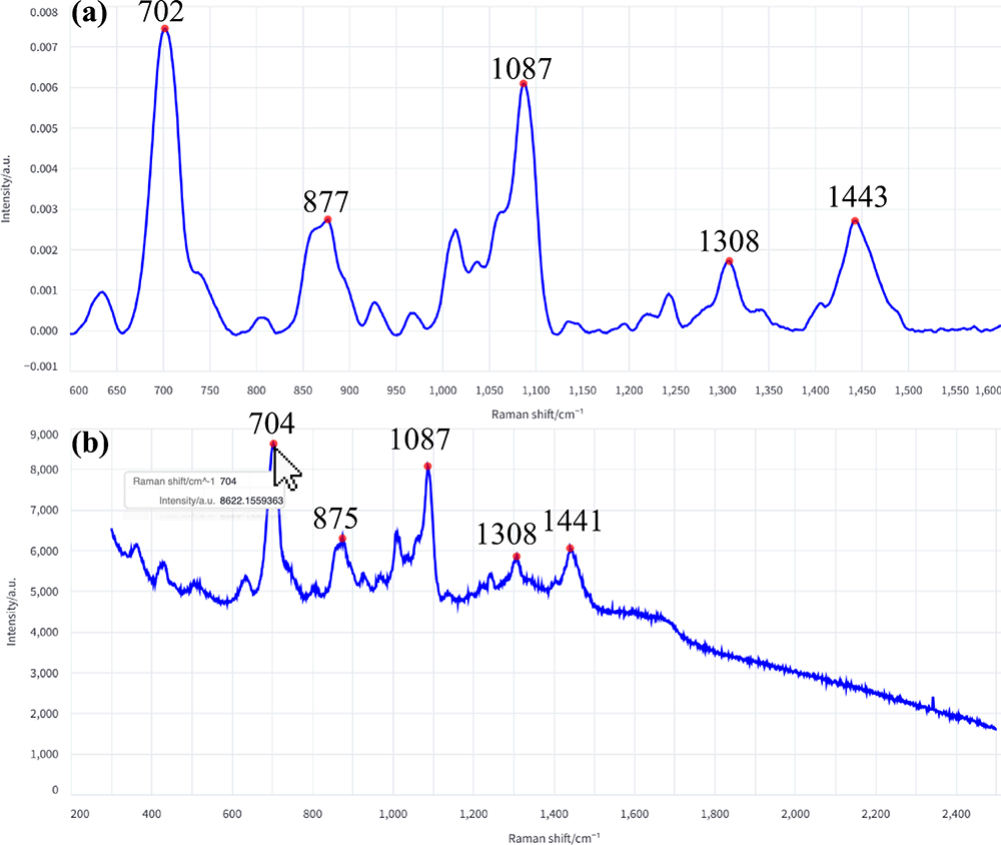
Peak identification on a representative (a) processed and (b) raw BSA spectrum. The red dots indicate the apex of the top five peaks selected based on prominence.

#### Average and Standard Deviation

The initial set of analytical tools focuses on descriptive statistics for assessing spectral reproducibility. In Figure 6a, the thick blue line denotes the mean spectrum computed from all processed spectra, whereas Figure 6b displays the corresponding standard deviation as a shaded region or separate trace, highlighting wavenumber regions with higher or lower variance among replicates.

**Figure 6:**
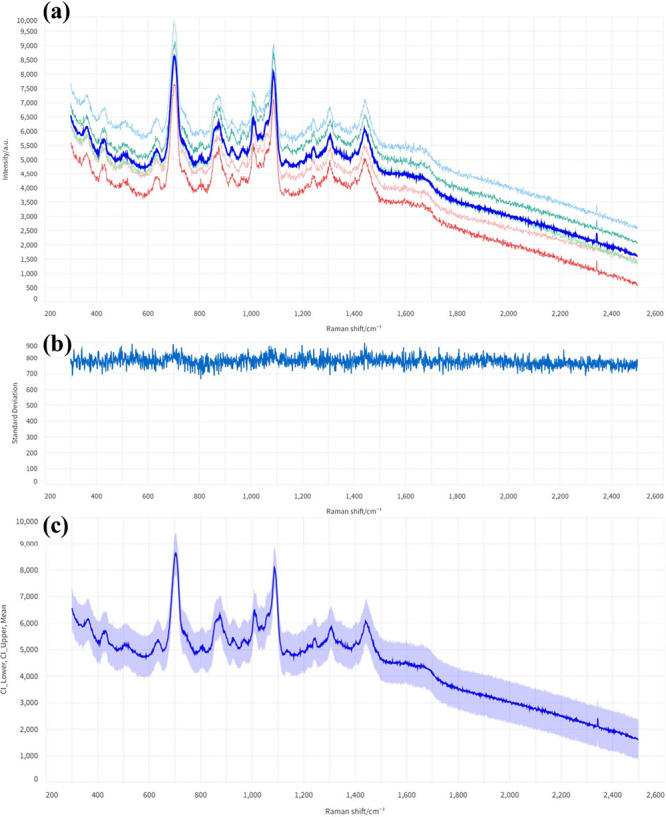
(a) Averaged spectrum, (b) corresponding standard deviation, and (c) confidence interval plot of the raw BSA spectra. In (c), the shaded region represents ±1 standard deviation around the mean (thick blue line), illustrating the wavelength-dependent variability within the data set.

#### Confidence Interval Plot

As part of the analytics toolkit, SpectraGuru provides a visualization of spectral reproducibility using a one-standard-deviation band around the mean spectrum. This representation highlights variability across replicates and helps assess the stability of the spectral features. In the raw BSA spectra (Figure 6c), the broad shaded region reflects substantial variability among measurements and the presence of background fluctuations. Typically, after preprocessing, this band becomes significantly narrower, indicating improved spectral consistency and reduced experimental noise.

#### Correlation Heatmap

SpectraGuru also provides a correlation heatmap to quantify spectral similarity across a data set. After preprocessing, as demonstrated in previous sections, pairwise Pearson correlation coefficients are computed for all spectra and organized into a symmetric correlation matrix. The heatmap displays these values, with both axes corresponding to spectrum indices and the averaged spectrum included as the final reference. Figure 7 illustrates the processed BSA spectra (Figure 4g) and their mean spectrum automatically computed, showing uniformly high correlations (≥0.95) across the matrix. Such consistently strong correlations indicate excellent reproducibility under identical experimental conditions.

**Figure 7:**
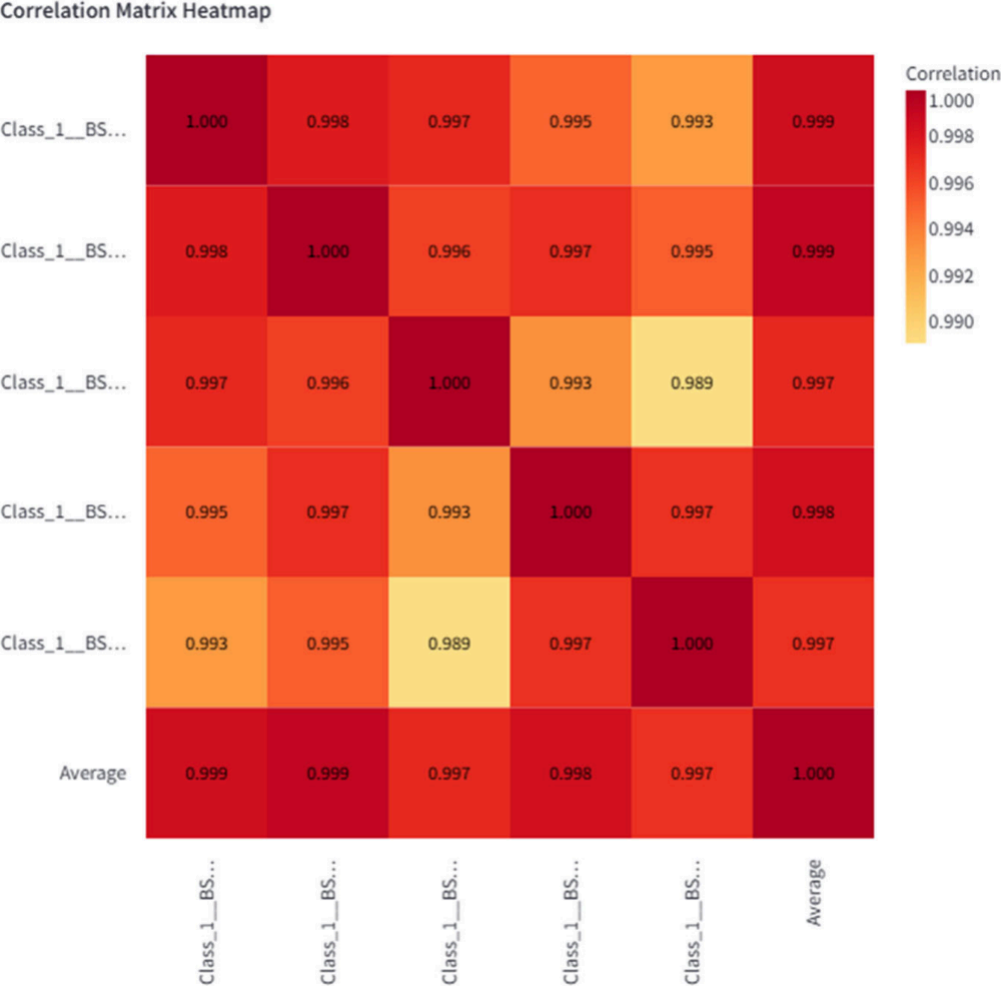
Correlation heatmap of the processed BSA data set.

#### Hierarchically Clustered Heatmap

SpectraGuru also incorporates hierarchical clustering to reveal structure within Raman and SERS data sets. Hierarchical Cluster Analysis (HCA) groups spectra based on pairwise similarity, making it useful for identifying subtle differences in peak positions or intensities that may reflect chemical or molecular variation. HCA operates by computing pairwise distances between spectra and applying an agglomerative clustering process that iteratively merges the most similar pairs. The resulting hierarchical tree, or dendrogram, visualizes relationships among spectra across varying levels of similarity. In SpectraGuru, HCA is implemented using Ward’s linkage method, which minimizes the increase in within-cluster variance at each step. This produces compact, well-defined clusters that are straightforward to interpret, allowing users to identify natural groupings, detect outliers, and evaluate reproducibility without prior labeling.

To demonstrate this functionality, HCA was applied to preprocessed spectra of 1,2-di­(4-pyridyl)­ethylene (BPE) and rhodamine 6G (R6G). Figure 8 provides an overview of this workflow. These data were retrieved directly from the SpectraGuru database through the Data Upload unit using the class-based query interface, where users provide a keyword, select matching entries, and load the corresponding spectra into the workspace. Figure 8a shows the spectra after preprocessing, highlighting the distinct Raman signatures of BPE and R6G. Once preprocessing is complete, the spectra are passed to the HCA module. Figure 8b presents the resulting hierarchically clustered heatmap. The top panel displays the dendrogram, and the bottom panel shows the spectral heatmap with Raman shift on the *y*-axis and individual spectra on the *x*-axis. Two dominant clusters emerge, corresponding to BPE and R6G, and the separation is reinforced by unmatched peak locations that distinguish the two analytes. Within each major cluster, additional second-level subclusters are visible, reflecting intraclass variability; for example, the R6G cluster separates into two groups with noticeable intensity differences.

**Figure 8:**
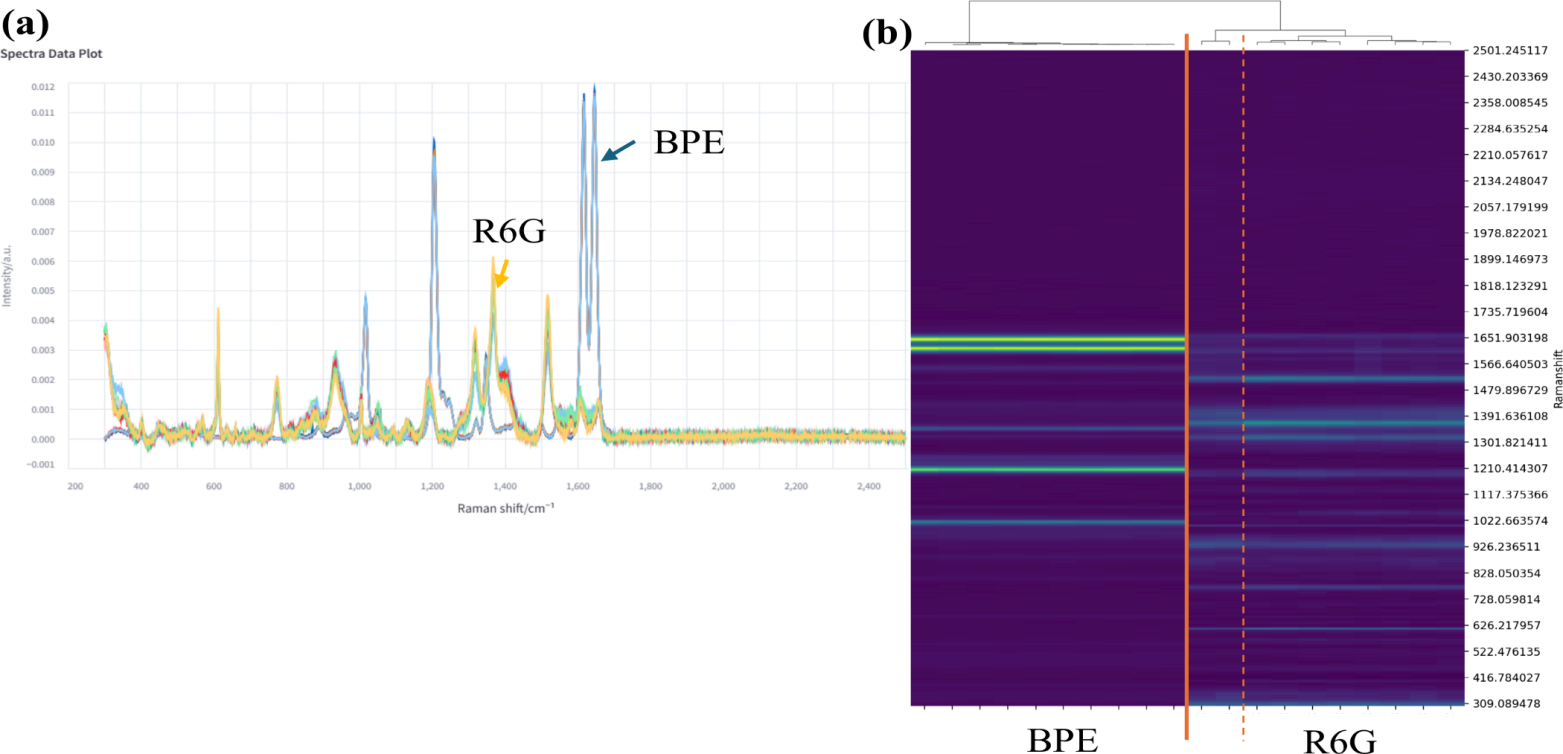
(a) Processed SERS spectra of BPE and R6G. (b) Hierarchical clustering dendrogram with accompanying heatmap, revealing two well-separated spectral clusters that align with the two analyte classes.

#### Principal Components Analysis (PCA)

PCA is a core dimensionality reduction method integrated into SpectraGuru for exploring high-dimensional spectral data sets. The algorithm is implemented using the PCA module from the scikit-learn library, which decomposes the data matrix into orthogonal components that successively capture the greatest variance. Each principal component (PC) represents a linear combination of the original spectral variables, with PC1 capturing the maximum variance, PC2 capturing the next highest variance orthogonal to PC1, and so forth. PCA was applied to the preprocessed BPE and R6G spectra in Figure 8a. Figure 9 summarizes the PCA results, displaying both the PC scatter plot and the cumulative variance curve that are generated simultaneously after the analysis is executed. The PC1–PC2 scatter plot (Figure 9a) shows two clearly separated clusters corresponding to BPE and R6G, demonstrating that the leading components successfully encode the major spectral differences and enable unsupervised discrimination based solely on vibrational features. Figure 9b presents the cumulative variance explained by successive principal components, where PC1 alone accounts for approximately 60% of the total variance, and the first six components collectively explain over 80% of the variance. This highlights PCA’s efficiency in reducing data dimensionality while retaining the majority of spectral information. Together, these visualizations demonstrate how PCA in SpectraGuru can be used to uncover underlying structures, identify discriminative features, and support exploratory analysis of Raman and SERS data.

**Figure 9:**
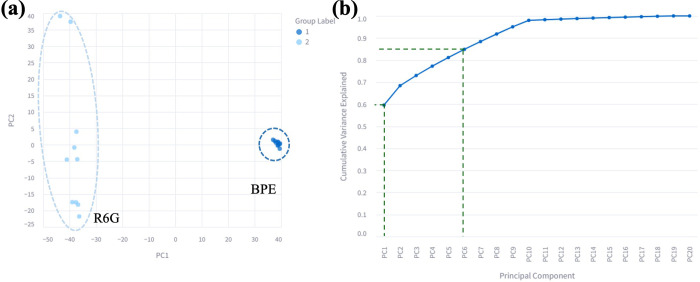
PCA of BPE and R6G spectral data. (a) Scattered plot of PC1 vs PC2. (b) Cumulative variance explained by the principal components.

#### T-Distributed Stochastic Neighbor Embedding (t-SNE) Dimensionality Reduction

SpectraGuru also includes t-Distributed Stochastic Neighbor Embedding (t-SNE), a nonlinear dimensionality reduction method designed to preserve local relationships within high-dimensional spectral data during projection to lower dimensions. Unlike linear methods such as PCA, t-SNE captures complex, nonlinear variations and highlights subtle spectral differences that may correspond to chemical or structural heterogeneity. For demonstration, t-SNE was applied to the same BPE and R6G data set (Figure 8a) using a perplexity of 3 and 500 optimization iterations by settings appropriate for small sample sizes. As shown in Figure 10, each point corresponds to an individual spectrum projected into two dimensions. The resulting embedding forms two well-separated clusters for BPE and R6G, consistent with the PCA results but with enhanced preservation of the local neighborhood structure.

**Figure 10:**
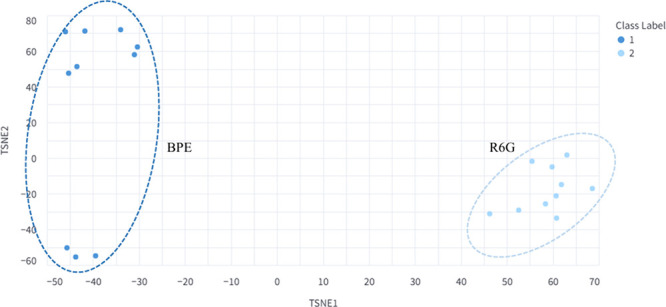
Two-dimensional t-SNE projection of the combined BPE and R6G data sets. Each point represents an individual spectrum, with distinct clustered regions (indicated by the dashed oval) corresponding to the two analytes.

## Conclusion and Future Work

This work presents SpectraGuru, an open-source, browser-based platform that provides a modular, end-to-end workflow for Raman and SERS analysis—from data upload and preprocessing to visualization and advanced analytics. Designed for accessibility and reproducibility, the platform enables users to convert raw spectra into clean, interpretable signals and derive insights through integrated statistical and multivariate tools. By unifying essential capabilities within a single interface and supporting seamless online updates, SpectraGuru lowers the barrier to high-quality spectral analysis while fostering community-driven development. As SpectraGuru integrates curated spectral databases, standardized metadata, and machine-learning pipelines, it is positioned to grow into a scalable, open-access cyberinfrastructure that supports reproducible and interoperable spectroscopy research. Early user feedback and adoption highlight the need for such a standardized, transparent environment. Future development will focus on improved scalability, greater modularity for algorithmic extensions, and sustained community engagement.

A major direction for future expansion is the introduction of advanced computational modules. One key feature under development is a deep learning unit that will allow users to interactively train, validate, and deploy neural network models directly within the platform. This capability will streamline machine-learning workflows for molecular sensing and diagnostics without requiring external programming environments or specialized hardware. Additional enhancements will extend the system’s scalability, modular architecture, and algorithmic flexibility, enabling users to plug in custom models, preprocessing functions, or feature-extraction pipelines.

As an open-source ecosystem, SpectraGuru is committed to growing through broad community participation. Contributions through GitHub will be encouraged from research groups worldwide, including new preprocessing routines, peak-analysis modules, spectral analytical tools, and machine-learning models. The internal spectral database will continue to expand to include published spectra across diverse instruments, materials, and experimental conditions accompanied by structured metadata compliant with FAIR principles. Community contributions follow clear guidelines and undergo automated quality checks or committee review, allowing the platform to evolve collaboratively while maintaining rigor and reproducibility.

To further strengthen community engagement, upcoming releases introduce structured contribution pathways for both data and algorithms. These will include standardized submission guidelines, automated quality checks, and contributor recognition via citation-ready data set identifiers and algorithm DOIs. Such infrastructure not only ensures academic credit for contributors but also promotes transparency, long-term reusability, and cross-laboratory comparability. In particular, the integration of recently proposed adaptive and parameter-free baseline correction algorithms is an active area of ongoing development.

In addition to these collaborative features, SpectraGuru can expand its role in spectroscopy education and training. Leveraging its open, installation-free interface, real-time visualization tools, and intuitive analytics pipeline, future versions will include curated educational tutorials, recommended preprocessing workflows, and guided learning modules. These resources aim to support classroom teaching, laboratory instruction, and self-paced learning, ultimately making advanced spectroscopy concepts more accessible to new practitioners.

In parallel, we are evaluating emerging technologies such as Web3DB^57^ to support decentralized, transparent, and secure data ownership infrastructures. By embracing these innovations and expanding its analytical toolkit, SpectraGuru aims to become a robust and adaptable resource for the spectroscopy community, accelerating discovery, reproducibility, and knowledge sharing across academic and industrial settings.

Planned future enhancements also include support for additional spectroscopy modalities beyond Raman and SERS, such as Fourier-transform infrared (FTIR) spectroscopy, X-ray photoelectron spectroscopy (XPS), UV–Vis, fluorescence, and mass spectrometry, enabling SpectraGuru to evolve into a comprehensive, multimodal analytical environment.

## Supplementary Material



## Data Availability

The source code of the application demonstrated in this manuscript is available at https://github.com/FengboMa/SpectraGuru_beta. The application is currently hosted at https://spectraguru.org/.

## Notes

Expanded documentation of the SpectraGuru platform, including implemented features and functionalities in SpectraGuru, supported data formats for manual data upload, database implementation detail, and mathematical formulations and algorithms used in SpectraGuru (PDF)
