# Supplementary material for: Comprehensive Open-Source Ecosystem for Raman and SERS Spectroscopy: Introducing SpectraGuru
Source: Anal Chem. 2026 Apr 6;98(15):11186–96. doi: 10.1021/acs.analchem.5c07799 (PMC13103934; doi:10.1021/acs.analchem.5c07799)
Supplement: Supplementary file 1 [file ac5c07799_si_001.pdf]

## Supporting Information

# Comprehensive Open-Source Ecosystem for Raman and SERS Spectroscopy: Introducing SpectraGuru

Fengbo Ma[1], Jiaheng Cui[1], Amit Kumar[2], Yanjun Yang[2], Xianyan Chen[3*], Yiping Zhao[2*]

[1] School of Electrical and Computer Engineering, College of Engineering, The University of Georgia, Athens, GA, USA 30602

[2] Department of Physics and Astronomy, The University of Georgia, Athens, GA, USA 30602

[3] Department of Epidemiology & Biostatistics, College of Public Health, The University of Georgia, Athens, GA, USA 30602

* Corresponding Author: E-mail: zhaoy@uga.edu; xychen@uga.edu

## Table of Contents

**S1. Implemented Features and Functionalities in SpectraGuru** ........................................................3
**S2. Supported Data Formats for Manually Data Upload** ....................................................................5
**S3. Database Implementation Detail**...........................................................................................7
**S4. Mathematical Formulations and Algorithms Used in SpectraGuru** ..........................................11

## S1. Implemented Features and Functionalities in SpectraGuru

**Table S1** provides a comprehensive summary of the preprocessing and analysis tools currently implemented in the SpectraGuru platform. Each feature is described in terms of its purpose, computational method, and implementation source.

**Table S1.** Summary of preprocessing and analysis features implemented in SpectraGuru. The table describes each feature, its functionality, and source of implementation

| Feature Name | Description | Source |
|---|---|---|
| ***Preprocessing Tools*** | | |
| Interpolation | Performs linear interpolation to align all spectra to a standardized wavenumber axis, rounding to integer values to facilitate data comparison. | Self-implemented |
| Crop | Crops the spectral data to retain only the wavenumber range specified by the user, focusing analysis on the region of interest. | Self-implemented |
| Despike (Auto) | Removes sharp cosmic ray spikes from spectral data by comparing each segment to a linear baseline and replacing outliers exceeding a defined threshold. | Self-implemented |
| Despike (Manual) | Refines spike removal by applying linear baseline correction only within a specified Raman shift range, allowing targeted cleanup of spectral regions prone to noise. | Self-implemented |
| Savitzky-Golay filter | Applies a Savitzky-Golay filter to smooth spectral data while preserving peak features, reducing noise without distorting the signal shape. | [1, 2] |
| 1D Fast Fourier Transform filter | Performs low-pass filtering on spectral data using Fast Fourier Transform (FFT), suppressing high-frequency noise while preserving the main signal shape, with optional padding to minimize edge effects. | Self-implemented[3] |
| Adaptive Iteratively Reweighted Penalized Least Squares for Baseline Fitting(airPLS) | Automatically corrects spectral baselines by adaptive iteratively reweighted penalized least squares airPLS algorithm to suppress background drift, without requiring prior knowledge of peak locations. | [4,5] |
| Modified Polynomial curve-fitting (ModPoly) | Performs baseline correction using an iterative modified polynomial fitting method, gradually refining the fit until minimal improvement is detected. | [6] |
| Gaussian-Lorentzian Function Fitting (GLFF) | Fits a mixed Gaussian-Lorentzian model to selected spectral regions to estimate and subtract the baseline, enabling cleaner signal analysis. | [7, 8] |
| Normalization by area | Normalizes the spectrum by its total area under the curve to ensure consistent intensity scaling across samples. | Self-implemented |
| Normalization by peak | Normalizes the spectrum by its maximum peak intensity to standardize signal amplitude across different spectra. | Self-implemented |
| MinMax Normalization | Scales the spectrum to a 0–1 range using min-max normalization, preserving relative intensity patterns while standardizing the value range. | Self-implemented |

| | | |
|---|---|---|
| Outlier Removal | Identifies and removes outlier spectra based on intensity deviation, Euclidean distance, and correlation with the average spectrum, improving data quality for downstream analysis. | Self-implemented |
| | ***Analysis Tools*** | |
| Average and Standard Deviation | Displays a combined analysis of the average Raman spectrum alongside original spectra, with a second panel showing the standard deviation across samples—offering a clear visual summary of signal consistency and variation. | Self-implemented |
| Confidence interval | Visualizes the spectral mean and its variability by plotting the average Raman spectrum with a shaded band representing one standard deviation, offering insights into signal consistency across samples. | Self-implemented |
| Correlation heatmap | Computes and visualizes the pairwise correlation matrix of spectral samples using Pearson correlation, highlighting linear similarity patterns between spectra for exploratory analysis or quality control. | Self-implemented |
| Peak Identification | Identifies peak positions in a spectrum using the SciPy find_peaks method, allowing customization of peak shape and prominence criteria for flexible and accurate feature detection. | [1] |
| Hierarchical Clustering Analysis (HCA) | Performs hierarchical clustering of spectral samples using Ward's method and visualizes the results as a dendrogram, revealing grouping patterns based on overall spectral similarity. | [1, 9] |
| Principal Component Analysis (PCA) | Performs PCA on standardized spectral data to reduce dimensionality and visualize patterns, including sample clustering, explained variance, and feature contributions across principal components. | [10, 11] |
| T-SNE | Applies t-SNE to reduce high-dimensional spectral data to two dimensions for visualization, revealing sample clustering and nonlinear patterns in a low-dimensional embedding. | [10, 12] |

## S2. Supported Data Formats for Manually Data Upload

Spectroscopy data are generated in diverse formats across instruments and vendors, and there is no standardized structure widely adopted in the field. Most proprietary software packages are optimized for their own file types, limiting cross-platform compatibility. To address this, SpectraGuru currently supports four widely used input formats that cover the majority of Raman and SERS instruments:

- Multi files: .txt files (two-column single spectrum files. common x)
- Multi files: .csv files (two-column single spectrum files. common x)
- Single file: .csv file (A tab-separated csv (tsv)file)
- Single file: .csv file (A comma-separated csv (csv)file)

Additional formats are under development for future releases.

The software architecture accommodates two primary input structures: (1) multiple individual files, each containing a single spectrum, and (2) a single consolidated file containing multiple spectra.

For multiple individual files, shown in **Figure S1**, each file must follow a two-column ASCII structure, such as .txt or .csv. The first column represents the independent variable (typically wavenumber, wavelength, or time), and the second column contains the dependent variable (intensity or absorbance). All files within a batch must share the same independent variable axis to maintain consistency during batch processing. For single consolidated files, SpectraGuru accepts data organized in a matrix structure where the first column represents the shared independent variable, and each subsequent column corresponds to a separate spectrum. Both comma-separated (.csv) and tab-separated (.tsv) formats are supported. All input data must contain strictly numerical values. The data parser automatically excludes one header row if present and rejects files containing text or null entries.

| Example Spectrum 1 | | Example Spectrum 2 | | Example Spectrum 3 | |
|---|---|---|---|---|---|
| 2501.088135 | 1652.905151 | 2501.088135 | 1553.588989 | 2501.088135 | 1408.161743 |
| 2500.383301 | 1550.041992 | 2500.383301 | 1642.264160 | 2500.383301 | 1383.332642 |
| 2499.678223 | 1574.618896 | 2499.678223 | 1556.886719 | 2499.678223 | 1432.761353 |
| 2498.973389 | 1624.009033 | 2498.973389 | 1538.908081 | 2498.973389 | 1421.894409 |
| 2498.268066 | 1545.752319 | 2498.268066 | 1666.292725 | 2498.268066 | 1474.846313 |
| 2497.562744 | 1538.415405 | 2497.562744 | 1634.123291 | 2497.562744 | 1467.520752 |
| 2496.857422 | 1531.080811 | 2496.857422 | 1640.949951 | 2496.857422 | 1456.653198 |
| 2496.151855 | 1679.666870 | 2496.151855 | 1591.076782 | 2496.151855 | 1399.722412 |
| 2495.446289 | 1548.305664 | 2495.446289 | 1651.053589 | 2495.446289 | 1385.326050 |
| 2494.740723 | 1569.312500 | 2494.740723 | 1707.468628 | 2494.740723 | 1406.359009 |
| 2494.034912 | 1668.234375 | 2494.034912 | 1671.776245 | 2494.034912 | 1434.468994 |
| 2493.328857 | 1572.351196 | 2493.328857 | 1575.892578 | 2493.328857 | 1469.652588 |
| 2492.622803 | 1596.884766 | 2492.622803 | 1724.352295 | 2492.622803 | 1462.335693 |
| 2491.916504 | 1713.455444 | 2491.916504 | 1702.834839 | 2491.916504 | 1370.056274 |

**Figure S1.** Examples of the two-column spectrum data structure, showing only a portion of each spectrum for illustration.

## S3. Database Implementation Detail

### S3.1 Metadata Schema and Database Infrastructure

Each spectral record in the SpectraGuru database is accompanied by a structured metadata file that ensures scientific reproducibility and interoperability across experiments. The metadata describe both the physical and procedural aspects of spectral acquisition, including the analyte name, buffer or solvent composition, measurement type, and instrument configuration. Key experimental parameters such as excitation wavelength, laser power, analyte concentration, and accumulation time are recorded in standardized units to enable quantitative comparison. Additional descriptors specify the experimental procedure, substrate type and material, and preparation conditions used during measurement. Notes and optional fields such as publication reference, operator identity, and preprocessing summary provide contextual details that help trace the provenance of each dataset and clarify how spectra were collected and processed.

All metadata are stored within a PostgreSQL-based infrastructure that organizes information into a standardized data table. Fields are grouped into four semantic categories: Analyte Information, Instrument or Acquisition Parameters, Substrate Information, and General Procedural Metadata. **Table S2** lists the variable names, data types, required status, and a concise definition for each field. When new data are being registered, the system validates the metadata fields and enforces consistent naming, numeric ranges, and date formats before insertion. Once accepted, the data are assigned a unique batch identifier, and the system updates the record with derived information such as the number of spectra in the batch and the preprocessing status. The database also maintains version tracking, ensuring that any modification to an existing dataset is time-stamped and logged for provenance.

**Table S2.** Structured metadata fields defined in the SpectraGuru database.

| Field Name | Data Type | Description |
|---|---|---|
| ***Analyte Information*** | | |
| Analyte Name | varchar | Name of the chemical or biological analyte measured in the spectrum. |
| Buffer Solution | varchar | Solvent or buffer matrix used during sample preparation. |
| Concentration | double precision | Numerical concentration value of the analyte. |
| Concentration Units | varchar | Units corresponding to the concentration value such as mol/L or mg/mL. |
| Preparation Conditions | text | Detailed description of how the analyte solution was prepared including sample handling steps. |
| ***Instrument/Acquisition Parameters*** | | |

| | | |
|---|---|---|
| Spectrum Input | varchar | Measurement type such as Raman or SERS. |
| Instrument Details | varchar | Instrument model or configuration used to acquire spectra. |
| Laser Wavelength | double precision | Excitation laser wavelength in nanometers. |
| Laser Power | double precision | Laser power in milliwatts applied during acquisition. |
| Accumulation | double precision | Integration or accumulation time in seconds for each measurement. |
| ***Substrate Information**** | | |
| Substrate Fabrication Procedure | text | Description of substrate fabrication steps such as deposition, coating, or nanostructure growth. |
| Substrate Type | varchar | General substrate classification such as glass, silicon, or polymer. |
| Substrate Material | varchar | Material composition of the SERS active layer. |
| ***General Procedural Metadata*** | | |
| Data Upload Date | date | Date when the batch was uploaded to the database. |
| Data Source | text | Publication, research group, etc. |
| Additional Note | text | Additional annotation provided by the user including comments on preprocessing or experiment context. |

Automated database triggers handle these background operations, including synchronization between raw and standardized tables, updating of upload timestamps, and management of preprocessing logs. The preprocessing history annotated under the Note field with each record summarizes the algorithms applied during spectral preparation, such as interpolation, smoothing, baseline removal, normalization, and outlier detection. These metadata entries allow users to reproduce the same data processing conditions or compare spectra processed under different parameter sets. The entire database infrastructure operates within the SpectraGuru backend, which integrates PostgreSQL with the Streamlit-based interface for data ingestion, query execution, and visualization. Data and plot export functions are embedded within the same framework, enabling researchers to retrieve both standardized spectra and associated metadata for downstream analysis or publication.

### S3.2 Size and Composition

The current composition of the SpectraGuru database is summarized in **Table S3** and **Table S4**, which together organize the dataset into standardized and raw experimental categories. The standard spectrum data table contains 44 standardized analyte entries, while the raw experiment spectrum data table captures 2975 raw experimental spectra recorded across 16 analytes. The total storage footprint is approximately 5.16 GB, with 5.63 MB attributed to the standardized dataset and 5.02 GB corresponding to raw experimental data. Across both tables, the database includes 60

analyte records, reflecting a broad diversity of chemical and biological materials. These datasets span 160 experiments, encompassing both curated reference measurements and a substantial collection of raw spectra. This structure provides a balanced foundation that integrates standardized spectral benchmarks with a larger body of experimental data, supporting robust data-driven analysis, model development, and reproducible research within the SpectraGuru ecosystem.

**Table S3.** Summary of standardized spectrum data recorded in the database; records are based on the database state as of Oct 14 2025 and may change as new data are added.

| Catalog | Analyte | Source |
|---|---|---|
| Chemical | Ampicillin | Self-collected |
| | Oxacillin | Self-collected |
| | Teicoplanin | Self-collected |
| | Vancomycin | Self-collected |
| | Metronidazole (MNZ) | Self-collected |
| | Ronidazole (RNZ) | Self-collected |
| | Carbendazim | [13] |
| | Chlorpyrifos | [13] |
| | 4-Mercaptobenzoic acid (4-MBA) | [14] |
| | Cytidine-5'-monophosphate (CMP) | [15] |
| | Guanosine-5'-monophosphate (GMP) | [15] |
| | Uridine-5'-monophosphate (UMP) | [15] |
| | Adenosine-5'-monophosphate (AMP) | [15] |
| | Melamine | [16] |
| Biomarker | Aflatoxin B1 (AFB1) | [17] |
| | Aflatoxin B2 (AFB2) | [17] |
| | Aflatoxin G1 (AFG1) | [17] |
| | Aflatoxin G2 (AFG2) | [17] |
| Dye | 1,2-di(4-pyridyl) ethylene (BPE) | Self-collected |
| Nucleic acid | let-7f DNA probe | [15] |
| | miR-224 DNA probe | [15] |
| | rev let-7f DNA probe | [15] |
| Virus | Human coronavirus 229E (CoV-229E) | [18] |
| | Human coronavirus NL63 (CoV-NL63) | [18] |
| | Human metapneumovirus (HMPV) | [18] |
| | Influenza A H1N1 | [18] |
| | Influenza A H3N2 | [18] |
| | Influenza B | [18] |
| | Adenovirus type 5 (Ad5) | [18] |
| | Human coronavirus OC43 (CoV-OC43) | [18] |
| | SARS-CoV-2 | [18] |
| | SARS-CoV-2 B1 | [18] |
| Bacteria | E. coli DH5α | [19] |
| | E. coli O157 | [19] |
| | Generic E. coli | [19] |
| | Salmonella typhimurium | [19] |
| | Staphylococcus aureus | [19] |

| | | |
|---|---|---|
| | Staphylococcus epidermidis | [19] |
| | Klebsiella pneumoniae (KP2) BAMC 07-18 | [20] |
| | Pseudomonas aeruginosa (PA2) PA 01 | [20] |
| | Pseudomonas putida (PP) USDACR | [20] |
| | Staphylococcus aureus (SA2) Xen 40 | [20] |
| | Citrobacter koseri (CK) USDACR | [20] |
| | Salmonella enterica (ST) USDACR | [20] |
| **Total** | | **44** |

**Table S4.** Summary of raw experimental spectrum data recorded in the database; counts are based on the database state as of Oct 14 2025 and may change as new data are added.

| Catalog | Analyte | # of spectrum | Source |
|---|---|---|---|
| Chemical | 2,3-Dihydroxybenzoic acid (2,3-DHBA) | 376 | [21] |
| | 2,5-Dihydroxybenzoic acid (2,5-DHBA) | 362 | [21] |
| | 5,5'-Dithiobis(2-nitrobenzoic acid) (DTNB) | 62 | [22] |
| | Melamine | 5 | [23] |
| | Urea | 4 | [24] |
| | Thiram | 14 | [25] |
| | Lactic acid | 5 | [25] |
| Dye | Beta-Carotene (β-Carotene) | 335 | [21] |
| | 1,2-di(4-pyridyl) ethylene (BPE) | 249 | [14] |
| | Rhodamine 6G (R6G) | 34 | [14] |
| | Para-Aminothiophenol (PATP) | 44 | [14] |
| Biomarker | Enterobactin | 360 | [21] |
| | Lipoteichoic acid (LTA) | 360 | [21] |
| | Pyocyanin | 360 | [21] |
| Experimental control | Substrate (control) | 6 | [26] |
| Virus | Adenovirus type 5 (Ad5) | 399 | [26] |
| **Total** | | **2975** | **16** |

## S4. Mathematical Formulations and Algorithms Used in SpectraGuru

### S4.1 Average and Standard Deviation

Average and standard deviation of a given spectra batch are calculated as:

$$\bar{I}_{SERS}(\Delta v_k) = \frac{1}{N}\sum_{i=1}^{N} I_i(\Delta v_k), \qquad k = (S1)$$

$$\sigma_{\mathrm{SERS}}(\Delta v_k) = \sqrt{\frac{1}{N-1}\sum_{i=1}^{N}\left(I_i(\Delta v_k) - \bar{I}_{SERS}(\Delta v_k)\right)^2}, \qquad k = (S2)$$

In these equations, $N$ denotes the total number of spectra in the dataset, while $\Delta v_k$ represents the wavenumber along the spectral axis, with k ranging from 1 to $M$. The term $I_i(\Delta v_k)$ denotes the intensity of the $i^{\mathrm{th}}$ spectrum at that wavenumber. The notation $\bar{I}_{SERS}(\Delta v_k)$ denotes the pointwise mean intensity computed across all spectra at each sampled wavenumber, and $\sigma_{\mathrm{SERS}}(\Delta v_k)$ provides the corresponding pointwise sample standard deviation.

### S4.2 Constructing confidence intervals

The mean intensity at each wavenumber $v$ was calculated together with the corresponding standard deviation across replicates. The blue line in Figure 6 represents the average spectrum, while the shaded region corresponds to the interval $\bar{I}_{SERS}(\Delta v_k) \pm \sigma_{\mathrm{SERS}}(\Delta v_k)$, providing a direct measure of reproducibility across spectra:

$$Band(\Delta v_k) = \bar{I}_{SERS}(\Delta v_k) \pm \sigma_{\mathrm{SERS}}(\Delta v_k), \qquad k = (S3)$$

### S4.3 Pearson correlation coefficient for the correlation heatmap

For two paired spectra $I_i(\Delta v_k)$ and $I_j(\Delta v_k)$ measured over the same set of wavenumbers from $k = 1,2,...,M$ the Pearson correlation coefficient $r_{ij}$ is defined as:

$$r_{ij} = \frac{\sum_{k=1}^{M}\left(I_i(\Delta v_k) - \overline{I_i}\right)\left(I_j(\Delta v_k) - \overline{I_j}\right)}{\sqrt{\sum_{k=1}^{M}\left(I_i(\Delta v_k) - \overline{I_i}\right)^2}\sqrt{\sum_{k=1}^{M}\left(I_j(\Delta v_k) - \overline{I_j}\right)^2}} (S4)$$

where $I_i(\Delta v_k)$ ($I_j(\Delta v_k)$) is the intensity of the $i^{\mathrm{th}}$ ($j^{\mathrm{th}}$) spectrum at wavenumber $\Delta v_k$, $\overline{I_i}$ is the mean intensity of spectrum $i$ across all wavenumbers, and $M$ is the total number of wavenumber points. The coefficient $r_{ij}$ ranges from -1 to +1, with values close to +1 indicating high similarity in spectral shape, values near 0 indicating no linear relationship, and negative values indicating inverse patterns.

### S4.4 Peak prominence definition used in peak identification

For a peak located at wavenumber $\Delta v_p$ with intensity $I(\Delta v_p)$, the prominence is defined as:

$$P(\Delta v_p) = I(\Delta v_p) - \max\left(I(\Delta v_L), I(\Delta v_R)\right)(S5)$$

where $I(\Delta v_L)$ and $I(\Delta v_R)$ are the wavenumbers of the lowest troughs on the left and right of the peak, bounded either by a higher neighboring peak or by the edge of the spectrum. This value represents the vertical distance from the peak apex to the higher of the two bounding minima.

### S4.5 Distance metrics and Ward's linkage formula for HCA

HCA begins with a pairwise distance matrix between all spectra, defined as:

$$d_{ij}(\Delta v_k) = \sqrt{\sum_{k=1}^{M} \left(I_i(\Delta v_k) - I_j(\Delta v_k)\right)^2}, \qquad k = 1,2,\ldots,M,(S6)$$

where $I_i(\Delta v_k)$ and $I_j(\Delta v_k)$ are the intensities of $i^{\text{th}}$ and $j^{\text{th}}$ spectra at wavenumber $\Delta v_k$, and $M$ is the total number of wavenumber points. Using these distances, agglomerative clustering iteratively merges spectra or clusters based on their similarity, producing a tree structure that reveals hierarchical relationships.

In SpectraGuru, HCA is implemented with Ward's linkage method, which merges clusters in a way that minimizes the increase in total within-cluster variance at each step. Formally, the Ward distance between two clusters $A$ and $B$ is given by:

$$D(A,B) = \frac{|A|\,|B|}{|A| + |B|}\ |\overline{I_A} - \overline{I_B}|^2, (S7)$$

where $|A|$and $|B|$denote the number of spectra in clusters $A$ and $B$, $\overline{I_A}$ and $I_B$ are the mean spectral vectors of the two clusters, and ‖·‖ is the Euclidean norm across all wavenumbers. This criterion favors merges that minimize the increase in within-cluster variance, thereby producing compact and interpretable groupings.

### S4.6 Covariance matrix, eigenvalue decomposition, and variance explanation in PCA

PCA facilitates the identification of dominant patterns and natural groupings, even in complex or noisy data.

For a dataset of Raman spectra represented by an $N \times M$ matrix $X$, where $N$ is the number of spectra and $M$ is the number of wavenumber points, the covariance matrix is given by:

$$C = \frac{1}{N-1} X^{\top} X. (S8)$$

PCA seeks the eigenvectors $\boldsymbol{w_k}$ and eigenvalues $\lambda_k$ of $C$:

$$C\boldsymbol{w_k} = \lambda_k \boldsymbol{w_k} (S9)$$

where each eigenvector $\boldsymbol{w_k}$ defines a principal component direction, and its corresponding eigenvalue $\lambda_k$ quantifies the variance explained by that component.

**S4.7 Conditional and joint probability definitions, KL divergence, and optimization procedure for t-SNE**

Given a batch of Raman or SERS spectra $\{I_1(\Delta v), I_2(\Delta v), ..., I_N(\Delta v)\}$, the similarity between two spectra $I_i$ and $I_j$ in the original high-dimensional wavenumber space is modeled by a conditional probability:

$$p_{j|i} = \frac{\exp\left(-\frac{\| I_{\mathrm{i}}(\Delta v) - I_{\square}(\Delta v) \|^2}{2\sigma_{\mathrm{i}}^2}\right)}{\sum_{k\neq i} \exp\left(-\frac{\| I_{\mathrm{i}}(\Delta v) - I_{\square}(\Delta v) \|^2}{2\sigma_{\mathrm{i}}^2}\right)}, (S10)$$

where $\| I_{\mathrm{i}}(\Delta v) - I_{\square}(\Delta v) \|^2$ denotes the squared Euclidean distance between the two spectra across all wavenumbers, and $\sigma_i$ is determined by the perplexity parameter that governs the effective neighborhood size. The joint probability is then symmetrized as:

$$p_{ij} = \frac{p_{j|i} + p_{i|j}}{2N}. (S11)$$

In the low-dimensional embedding, each spectrum is represented by a coordinate $y_i$. Pairwise similarities are modelled using a Student's $t$ distribution with one degree of freedom:

$$q_{ij} = \frac{\left(1 + \| y_i - y_j \|^2\right)^{-1}}{\sum_{k\neq l} (1 + \| y_k - y_l \|^2)^{-1}} (S12)$$

The embedding is optimized by minimizing the Kullback–Leibler (KL) divergence between the high-dimensional distribution $P$ and the low-dimensional distribution $Q$:

$$KL(P \| Q) = \sum_{i\neq j} p_{ij} \log \frac{p_{ij}}{q_{ij}} (S13)$$

Each $y_i$ thus represents the projection of spectrum $I_{\mathrm{i}}(\Delta v)$ into a 2D space. Spectra that are close together in the original high-dimensional space will tend to be mapped nearby in the low-dimensional embedding.

## References


(1) Virtanen, P.; Gommers, R.; Oliphant, T. E.; Haberland, M.; Reddy, T.; Cournapeau, D.; Burovski, E.; Peterson, P.; Weckesser, W.; Bright, J.; van der Walt, S. J.; Brett, M.; Wilson, J.; Millman, K. J.; Mayorov, N.; Nelson, A. R. J.; Jones, E.; Kern, R.; Larson, E.; Carey, C. J.; Polat, İ.; Feng, Y.; Moore, E. W.; VanderPlas, J.; Laxalde, D.; Perktold, J.; Cimrman, R.; Henriksen, I.; Quintero, E. A.; Harris, C. R.; Archibald, A. M.; Ribeiro, A. H.; Pedregosa, F.; van Mulbregt, P. SciPy 1.0: Fundamental Algorithms for Scientific Computing in Python. *Nat. Methods* **2020**, *17* (3), 261–272. https://doi.org/10.1038/s41592-019-0686-2.

(2) Savitzky, Abraham.; Golay, M. J. E. Smoothing and Differentiation of Data by Simplified Least Squares Procedures. *Anal. Chem.* **1964**, *36* (8), 1627–1639. https://doi.org/10.1021/ac60214a047.

(3) Zhang, X.; Jiang, S. Application of Fourier Transform and Butterworth Filter in Signal Denoising. In *2021 6th International Conference on Intelligent Computing and Signal Processing (ICSP)*; 2021; pp 1277–1281. https://doi.org/10.1109/ICSP51882.2021.9408933.

(4) Zhang, Z.-M.; Chen, S.; Liang, Y.-Z. Baseline Correction Using Adaptive Iteratively Reweighted Penalized Least Squares. *The Analyst* **2010**, *135* (5), 1138–1146. https://doi.org/10.1039/b922045c.

(5) Cui, J.; Chen, X.; Zhao, Y. Beyond Traditional airPLS: Improved Baseline Removal in SERS with Parameter-Focused Optimization and Prediction. *Anal. Chem.* **2025**, *97* (30), 16211–16218. https://doi.org/10.1021/acs.analchem.5c01253.

(6) Lieber, C. A.; Mahadevan-Jansen, A. Automated Method for Subtraction of Fluorescence from Biological Raman Spectra. *Appl. Spectrosc.* **2003**, *57* (11), 1363–1367.

(7) Yang, Y.; Xu, B.; Murray, J.; Haverstick, J.; Chen, X.; Tripp, R. A.; Zhao, Y. Rapid and Quantitative Detection of Respiratory Viruses Using Surface-Enhanced Raman Spectroscopy and Machine Learning. *Biosens. Bioelectron.* **2022**, *217*, 114721. https://doi.org/10.1016/j.bios.2022.114721.

(8) Yang, Y.; Xu, B.; Haverstick, J.; Ibtehaz, N.; Muszyński, A.; Chen, X.; Chowdhury, M. E. H.; Zughaier, S. M.; Zhao, Y. Differentiation and Classification of Bacterial Endotoxins Based on Surface Enhanced Raman Scattering and Advanced Machine Learning. *Nanoscale* **2022**, *14* (24), 8806–8817. https://doi.org/10.1039/D2NR01277D.

(9) Ward Jr., J. H. Hierarchical Grouping to Optimize an Objective Function. *J. Am. Stat. Assoc.* **1963**, *58* (301), 236–244. https://doi.org/10.1080/01621459.1963.10500845.

(10) Pedregosa, F.; Varoquaux, G.; Gramfort, A.; Michel, V.; Thirion, B.; Grisel, O.; Blondel, M.; Müller, A.; Nothman, J.; Louppe, G.; Prettenhofer, P.; Weiss, R.; Dubourg, V.; Vanderplas, J.; Passos, A.; Cournapeau, D.; Brucher, M.; Perrot, M.; Duchesnay, É. Scikit-Learn: Machine Learning in Python. arXiv June 5, 2018. https://doi.org/10.48550/arXiv.1201.0490.

(11) Hotelling, H. Analysis of a Complex of Statistical Variables into Principal Components. *J. Educ. Psychol.* **1933**, *24* (6), 417–441. https://doi.org/10.1037/h0071325.

(12) Maaten, L. van der; Hinton, G. Visualizing Data Using T-SNE. *J. Mach. Learn. Res.* **2008**, *9* (86), 2579–2605.

(13) Wang, T.; Dong, P.; Zhu, C.; Gao, W.; Sha, P.; Wu, Y.; Wu, X. Fabrication of 2D Titanium Carbide MXene/Au Nanorods as a Nanosensor Platform for Sensitive SERS Detection. *Ceram. Int.* **2021**, *47* (21), 30082–30090. https://doi.org/10.1016/j.ceramint.2021.07.184.

(14) Wang, C.; Li, P.; Wang, J.; Rong, Z.; Pang, Y.; Xu, J.; Dong, P.; Xiao, R.; Wang, S. Polyethylenimine-Interlayered Core–Shell–Satellite 3D Magnetic Microspheres as Versatile

SERS Substrates. *Nanoscale* **2015**, *7* (44), 18694–18707. https://doi.org/10.1039/C5NR04977F.

(15) Abell, J. L.; Garren, J. M.; Driskell, J. D.; Tripp, R. A.; Zhao, Y. Label-Free Detection of Micro-RNA Hybridization Using Surface-Enhanced Raman Spectroscopy and Least-Squares Analysis. *J. Am. Chem. Soc.* **2012**, *134* (31), 12889–12892. https://doi.org/10.1021/ja3043432.

(16) Du, X.; Chu, H.; Huang, Y.; Zhao, Y. Qualitative and Quantitative Determination of Melamine by Surface-Enhanced Raman Spectroscopy Using Silver Nanorod Array Substrates. *Appl. Spectrosc.* **2010**, *64* (7), 781–785. https://doi.org/10.1366/000370210791666426.

(17) Wu, X.; Gao, S.; Wang, J.-S.; Wang, H.; Huang, Y.-W.; Zhao, Y. The Surface-Enhanced Raman Spectra of Aflatoxins: Spectral Analysis, Density Functional Theory Calculation, Detection and Differentiation. *Analyst* **2012**, *137* (18), 4226–4234. https://doi.org/10.1039/C2AN35378D.

(18) Yang, Y.; Xu, B.; Murray, J.; Haverstick, J.; Chen, X.; Tripp, R. A.; Zhao, Y. Rapid and Quantitative Detection of Respiratory Viruses Using Surface-Enhanced Raman Spectroscopy and Machine Learning. *Biosens. Bioelectron.* **2022**, *217*, 114721. https://doi.org/10.1016/j.bios.2022.114721.

(19) Chu, H.; Huang, Y.; Zhao, Y. Silver Nanorod Arrays as a Surface-Enhanced Raman Scattering Substrate for Foodborne Pathogenic Bacteria Detection. *Appl. Spectrosc.* **2008**, *62* (8), 922–931. https://doi.org/10.1366/000370208785284330.

(20) Wu, X.; Huang, Y.-W.; Park, B.; Tripp, R. A.; Zhao, Y. Differentiation and Classification of Bacteria Using Vancomycin Functionalized Silver Nanorods Array Based Surface-Enhanced Raman Spectroscopy and Chemometric Analysis. *Talanta* **2015**, *139*, 96–103. https://doi.org/10.1016/j.talanta.2015.02.045.

(21) Kumar, A.; Islam, M. R.; Zughaier, S. M.; Chen, X.; Zhao, Y. Precision Classification and Quantitative Analysis of Bacteria Biomarkers via Surface-Enhanced Raman Spectroscopy and Machine Learning. *Spectrochim. Acta. A. Mol. Biomol. Spectrosc.* **2024**, *320*, 124627. https://doi.org/10.1016/j.saa.2024.124627.

(22) Zhu, C.; Dong, P.; Wu, X. Amplification-Free DNA Biosensing by Surface-Enhanced Raman Scattering Based on Au Nanobipyramids Decorated Ag Nanorod Array. In *2023 IEEE 18th International Conference on Nano/Micro Engineered and Molecular Systems (NEMS)*; 2023; pp 165–169. https://doi.org/10.1109/NEMS57332.2023.10190901.

(23) Wang, T.; Gao, W.; Zhu, C.; Sha, P.; Wu, Y.; Wu, X.; Dong, P. Au Nanoparticle/Graphene Oxide Composites Deposited on Au Nanorod Arrays as Substrates for Surface-Enhanced Raman Scattering Sensing. *ACS Appl. Nano Mater.* **2022**, *5* (1), 1086–1094. https://doi.org/10.1021/acsanm.1c03728.

(24) Xiong, S.; Wang, C.; Zhu, C.; Dong, P.; Wu, X. Dual Detection of Urea and Glucose in Sweat Using a Portable Microfluidic SERS Sensor with Silver Nano-Tripods and 1D-CNN Model Analysis. *ACS Appl. Mater. Interfaces* **2024**, *16* (48), 65918–65926. https://doi.org/10.1021/acsami.4c14962.

(25) Xiong, S.; Wang, C.; Wang, T.; Zhu, C.; Dong, P.; Wu, X. Label-Free Detection of Sweat Biomarkers Using AuNRAs-Based SERS-Digital Microfluidic Sensor. *Chem. Eng. J.* **2025**, *510*, 161849. https://doi.org/10.1016/j.cej.2025.161849.

(26) Yang, Y.; Cui, J.; Kumar, A.; Luo, D.; Murray, J.; Jones, L.; Chen, X.; Hülck, S.; Tripp, R. A.; Zhao, Y. Multiplex Detection and Quantification of Virus Co-Infections Using Label-

Free Surface-Enhanced Raman Spectroscopy and Deep Learning Algorithms. *ACS Sens.* **2025**, *10* (2), 1298–1311. https://doi.org/10.1021/acssensors.4c03209.
